# 

**Published:** 1808-11

**Authors:** 


### Medical and Phyfical Journal.

VOL. XX.] NOVEMBER 1808. [NO. 117.

*Printed for R. PHILLIPS, by W. Thorne, Red Lion Court, Fleet Street, London*

*Our readers will observe by the title page, that the name of Dr. Adams is substituted for that of Dr. Batty. The department of the profession in which Dr. Batty is chiefly engaged, frequently rendering it impossible for him to lend his assistance when it was most wanted, he has found it necessary to decline the engagement altogether.*

*The present Editors, in requesting the continuance of the favours of their Correspondents, are aware that some objections have been made to the admission of certain controversial papers, and to others which have appeared to be of minor importance. The first class it has been the business of the Editors for some time to discourage. Respecting the second, it should be recollected, that many papers, apparently unimportant, have given rise to discussions highly valuable in their result. All importance is relative, and that which is familiar to some readers, is new and interesting to others. However, as this objection is likely to exist among our best informed readers, we hope they will afford the true remedy, by furnishing us with valuable communications of their own.*

*The critical department, which has always been distinct from the editorial, will remain in the same hands as heretofore. In this part of the Journal, it has been the wish of the Editors that unnecessary se-*

*verity should be avoided; that the spirit of the works reviewed should be transfused into their pages; and that all imperfections should be pointed out, in a manner which may convince the author that they really exist. If, however, any author conceives himself mis-understood, or treated with undue severity, the Journal affords a fair opportunity to reply.*

*In acknowledging the liberality with which this undertaking has been supported, during a period of eleven years, the present Editors promise an increase of diligence proportionate to the great encouragement with which the work has been honoured.*

*It may be presumed, that many professional gentlemen, who have become stationary during the progress of this Journal, will wish to include in their Libraries a work which forms a complete Medical History of the last ten years. To accommodate such, and to remove every impediment to the increasing circulation of the work, the Proprietor has consented for the next two months, or during the current year, to deliver complete sets of* NINETEEN VOLUMES *at the reduced price of* TEN POUNDS IN BOARDS, *instead of the usual price of* THIRTEEN GUINEAS; *but, to make up for such a diminution of his own profits, it will be necessary for those who wish to become purchasers on these terms, to address themselves directly to him, and their orders will meet with prompt attention.*

NEW MEDICAL PUBLICATIONS.

Observations on the Egyptian Ophthalmia, and Ophthalmia Purulenta, as it has appeared in England; by William Thomas, Member of the Royal College of Surgeons, and Assistant Surgeon in the Royal Veteran Battalion, 2s. 6d.

Remarks on the Frequency and Fatality of different Diseases, particularly on the progressive Increase of Consumption, with Observations on the Influence of the Seasons on Mortality; by William Woolcombe, M. D. 8vo. 6s.

An Exposure and Refutation of various Misrepresentations, published by Dr. M'Gregor and Dr. Jackfon, in Three Letters to the Commissioners of Military Enquiry, interspersed with Facts and Observations concerning Military Hospitals, and Medical Arrangements for Armies; by E. N. Bancroft, M. D. 4s.

